# Systemic Inflammation in Pregnant Women With Periodontitis and Preterm Prelabor Rupture of Membranes: A Prospective Case-Control Study

**DOI:** 10.3389/fimmu.2019.02624

**Published:** 2019-11-07

**Authors:** Stefan Mohr, Sofia K. Amylidi-Mohr, Pascale Stadelmann, Anton Sculean, Rutger Persson, Sigrun Eick, Daniel V. Surbek

**Affiliations:** ^1^Department of Obstetrics and Gynecology, Inselspital, Bern University Hospital, University of Bern, Bern, Switzerland; ^2^Department of Periodontology, School of Dental Medicine, University of Bern, Bern, Switzerland; ^3^Oral Health Sciences, Division of Health Sciences, Research Professor, University of Washington, Seattle, WA, United States

**Keywords:** adverse pregnancy outcome, cytokines, inflammatory mediators, periodontal inflammation, periodontopathogenic bacteria, preterm birth, PPROM, preterm premature rupture of membranes

## Abstract

**Aims:** Periodontal disease is associated with adverse pregnancy outcome, but the underlying pathophysiologic mechanism is still unknown. In this prospective, longitudinal, non-interventional case-control study, 45 women with preterm premature rupture of membranes and 26 controls with uncomplicated pregnancies were examined at three time-points (T1: 20–34 weeks of gestations; T2: within 48 h after delivery; T3: 4–6 weeks post partum). Examinations included subgingival, blood, vaginal, and placenta sampling for microbiologic, cytokine, and histology assessment. Objective of this study was to test the hypothesis that systemic inflammatory changes and not specific bacteria are predominantly involved in the association between periodontal disease and adverse pregnancy outcome.

**Results:** Demographic data and gestational age at T1 were comparable between groups. While there was no correlation between vaginal and gingival fluid microbiome, cytokine levels in the assessed compartments differed between cases, and controls. Vaginal smears did not show a higher rate of abnormal flora in the cases at the onset of preterm premature rupture of membranes. Number and variety of bacteria in the case group placental membranes and vagina were higher, but these bacteria were not found in membranes at birth.

**Conclusions:** On the basis of our results we speculate that an inflammatory pathway sequentially involving periodontal tissue, maternal serum, and finally vaginal compartment contributes to the underlying pathomechanism involved in preterm premature rupture of membranes associated with periodontitis.

## Introduction

Preterm birth (PTB) is the leading cause of perinatal morbidity and mortality in developed countries (1). About 10% of all live births worldwide are preterm and account for 28–75% of all perinatal deaths and over 50% of all severe developmental disorders in children worldwide (1). Prevention of PTB would have an extensive impact on public health and understanding its pathogenesis would pave the way for prevention strategies.

PTB is associated with preterm labor in 40–45%, with preterm prelabor rupture of membranes (PPROM) in 25–30%, and with maternal or fetal indications in 30–35% (1, 2). PPROM occurs in 3% of pregnancies (2).

Ascending (asymptomatic) intrauterine infections cause an inflammatory decidual activation resulting in preterm labor or PPROM (3–5). Albeit this is believed to be the primary cause of PPROM (1, 6, 7), to what extent remote infections contribute to PTB and specifically PPROM is not known (1, 8, 9). Many of the risk factors for PTB culminate in increased systemic inflammation which might as well result in decidual activation (1).

Periodontal disease is known to be related to systemic inflammation in other diseases (10). Periodontitis is an inflammatory disease destroying tooth-supporting connective tissue and bone. It has been linked to adverse pregnancy outcome (APO) like PTB, low birth weight and preeclampsia (8, 11–17), but this association is inconsistently reported to exist or not to exist (18, 19).

The pathophysiologic mechanism of a potential association between APO and periodontal disease remains to be elucidated (8, 20). Possibly a common pathway of increased systemic inflammation is responsible (8, 21), since intrauterine infection with oral flora in PTB patients is rare (22) and bacteria are found in the majority of elective cesarean membranes at term, hence the presence of bacteria may not be sufficient to cause PTB (1, 23).

Recent findings challenge the view that ascending infections are the primary cause for PTB because the placental microbiome profile is much more akin to the oral microbiome than to the lower genital tract (24). Furthermore, bacteria detected in the PTB placenta are not typically found in the lower genitourinary tract (24). Intrauterine infections causing PTB may originate from the mother's mouth rather than the vagina and the oral cavity is a major reservoir for microbial infections (25, 26). Nevertheless, it is unclear whether the organisms need to enter fetal tissue or whether inflammatory substances are sufficient to potentiate PTB (27).

Microorganisms activate the immune system to release inflammatory markers and prostaglandins stimulating uterine contractility and matrix-degrading enzymes which lead to PPROM (1, 7). Increased levels of inflammatory mediators in gingival crevicular fluid (GCF) have been found in women with APO and pro-inflammatory cytokines might be able to precipitate labor (28–31).

The study of clinical parameters, bacteria and cytokines in three different body compartments (mouth, vagina, blood) might help to understand the relation between remote infections and PTB. Therefore, the aim of the present study was to investigate inflammatory markers in different body compartments in women with PPROM compared with healthy pregnant women to test the hypothesis that systemic inflammatory changes and not specific bacteria are predominantly involved in the association between periodontal disease and APO.

## Materials and Methods

### Study Design and Patient Selection

This longitudinal, non-interventional, prospective case-control study was designed and conducted as a collaboration of the Department of Obstetrics and Gynecology and the Department of Periodontology (University of Bern). The study protocol was approved by the Ethics Committee of the Canton of Bern (Nr. 091/10), and all participating women gave written informed consent.

Participants were enrolled in the Department of Obstetrics and Gynecology from November 2011 to August 2013 when either presenting with PPROM or during regular pregnancy visits. Cases were defined as women with PPROM between 20 0/7 and 34 0/7 weeks of gestation, and controls had uneventful pregnancies at the time of inclusion and were recruited in the same time frame (timepoint T1).

Cases and controls were examined at three distinct time points T1, T2, T3 (T1: after inclusion; T2: within 48 h after delivery; T3: 4–6 weeks post partum). Women missing two out of three examinations were defined as dropouts. Each exam included

1. Oral investigation: Periodontal Screening Index (PSI), collection of gingival crevicular fluid [GCF; CRP, cytokines (IL-1b, IL-6, IL-8, IL-10)], subgingival bacterial sampling, Multiplex-PCR
2. Blood samples: CRP, blood count, cytokines (IL-1b, IL-6, IL-8, IL-10)
3. Vaginal exam: vaginal and cervical swabs [gram stain (Nugent-Score), microbiologic culture and PCR], CRP, cytokines (IL-1b, IL-6, IL-8, IL-10), Multiplex-PCR
4. Additionally, at T2 placental membranes were examined microbiologically [gram stain (Nugent-Score), microbiologic culture and PCR] and histopathologically.

Women with PPROM received antibiotic treatment (clindamycin or amoxicillin/clavulanic acid if group B streptococcal status was positive or unknown) for 10 days. Tocolysis was administered. Antenatal glucocorticoids (betamethasone 12 mg i.m., repeated once after 24 h) were given to promote fetal lung maturation. Samples were taken before antibiotic, tocolytic, and glucocorticoid treatment on initial exam after admission. Labor was induced or cesarean section done at 34 0/7 weeks of gestation if women did not develop spontaneous labor, persistent vaginal bleeding, signs of amnion infection, or non-reassuring fetal status.

Statistical power analysis based on data including 185 patients at 6 months post-partum (32). A 50% difference in bacterial load between women delivering preterm and controls was anticipated. Based on a significance level of *p* < 0.01 and 1-β = 0.85 we assumed to need 40 women in each group to declare a difference.

### Oral Investigation

For microbiological sampling subgingival plaque was collected from each first molar in all quadrants with endodontic paper points (ISO 055, Dentsply Maillefer, Montigny Le Bretonneux, France, www.dentsply.fr) inserted into the gingival crevice for 15 s. Paper points pooled and stored at −20°C until assayed for presence of periodontopathogens. The PSI (Periodontal Screening Index) was recorded. GCF (Gingival crevicular fluid) samples were taken by sterile paper strips (Periopaper, Oraflow Inc., Smithtown, NY, USA, www.oraflow.com) and stored at −80°C until assayed. The oral investigations' results have been published elsewhere (12).

### Vaginal and Peripheral Blood Analysis, Histopathology

Swabs were taken from the vagina and cervix, respectively, during speculum exam for routine microbiological culture, PCR, and gram stain and stored in the appropriate media. Blood samples were examined in the central laboratory and histopathologic assessment was provided from the Department of Pathology (University Hospital Bern).

### Microbiologic Analysis

Multiplex-PCR: DNA was extracted by using the Chelex method (12). For detection of periodontopathogens the microIDent®plus11 test (Hain Lifescience, Nehren, Germany, www.hain-lifescience.de) was used according to the manufacturer's description. The test is able to identify 11 periodontopathogenic bacterial species after two PCR runs and a subsequent reverse hybridization (12).

Vaginal samples were assessed via conventional microbiological analysis (cultures including aerobes and anaerobes, *Listeria* and *Candida* sp.) and PCR (Chlamydia, Mycoplasma, Ureaplasma, and Treponema pallidum). Bacterial vaginosis (BV) was diagnosed in gram stain samples according to Nugent criteria and smears were categorized as abnormal according to Donders et al. (33).

### Analysis of Inflammatory Mediators

Before analyzation, samples (GCF, blood serum, vaginal fluid) were eluted at 4°C overnight into 750 μl phosphate-buffered saline containing proteinase inhibitors (Sigma-Aldrich, St. Louis, MO, USA, www.sigmaaldrich.com). From the eluates, IL-1b, IL-6, IL-8, IL-10, and CRP were determined by using commercially available enzyme-linked immunosorbent assay (ELISA) kits (R&D Systems Europe Ltd., Abingdon, UK, www.rndsystems.com). The detection levels of the kits were 2 pg (CRP: 10 pg).

### Data Analysis

Demographic data, obstetric outcomes and laboratory results were compared with the student's *t*-test and the Mann-Whitney-*U*-Test, respectively. The Mann-Whitney-*U* Rank Sum Test was used comparing medians if the normality test (Kolmogorov-Smirnov) failed or if the normality test passed but the equal variance test (Levene Median test) failed. If both tests passed the student's *t*-test was used for comparing means. Qualitative data were analyzed with Fisher's Exact Test for independent groups. The statistical tests used are specified in the respective tables. Statistical analysis was performed by using Graph Pad Prism 6 (GraphPad Software, Inc., La Jolla, CA, USA). The level of significance was set at *p* = 0.05.

## Results

Seventy-one women were included in the study analysis of whom 45 were PPROM-cases and 26 controls. In the test group four women dropped out because they did not prove to have ruptured membranes (*n* = 2), did not deliver in our facility or were not followed-up according to the protocol. In the control group 11 women dropped out as they delivered in other hospitals (*n* = 3), developed chorioamnionitis (*n* = 2), had a severe pre-eclampsia, intrauterine death, preterm contractions, or refused further participation in the study (*n* = 3).

Study population and controls were comparable as they did not differ in regard with age, race, BMI, parity, and preexisting conditions (Table 1).

**Table 1:** Demographic data, preexisting conditions, previous and current pregnancies.

		**Cases (*n* = 45)**	**Controls (*n* = 26)**	***p*=**	
Age	(years; Median, Range)	35.0 (24–48)	35.0 (25–42)	0.608^*^	n.s.
Race	(% caucasian)	91	81	0.272°	n.s.
BMI	(kg/m^2^; Median, Range)	22.2 (16.1–44.3)	22.2 (18–40.4)	0.989^**^	n.s.
Primipara	(%)	58	58	1.000°	n.s.
Preexisting conditions		(*n* = )			
- Systemic diseases		5	4		
- Hypertensive disease		1	0		
- Obesity		3	3		
- HIV-positive		1	0		
Previous pregnancies					
- Miscarriage		15	8		
- Induced abortion		3	2		
- Preterm delivery		1	1		
- Intrauterine fetal death		1	1		
- Chorioamnionitis		1	0		
- Preeclampsia		1	1		
- PPROM		1	2		
- GTD		0	1		
Current pregnancy					
- Premature labor		26	0		
- Amnion infection syndrome		9	0		
- Vaginal bleeding		4	2		
- Polyhydramnios		1	0		
- Pregnancy-induced hypertension		0	2		
- Gestational diabetes		0	1		
- Preeclampsia		0	1		

* t-test;

** *Mann-Whitney-U-Test; °Fisher's Exact test; n.s., not statistically significant*.

Characteristics of previous and current pregnancies were similar in both groups, too, except from premature labor, and amnion infection syndrome which were understandably more frequent in the test group (Table 1).

As expected, pregnancies lasted significantly shorter and birth weights were significantly lower in the case group. There was only a tendency toward a higher rate of cesarean deliveries in the case group which was not statistically significant (Table 2).

**Table 2:** Current pregnancy.

		**Cases (*n* = 45)**	**Controls (*n* = 26)**	***p* =**	
Gestational weeks at PPROM/study inclusion	Median	31 + 0	28 + 1	0.033^**^	
	Range	22 + 5–35 + 1	21 + 1–35 + 1		
Gestational weeks at delivery	Median	32 + 2	39 + 1	<0.001^**^	
	Range	25 + 2–35 + 1	35 + 2–41 + 3		
Complications at delivery	*n* =				
- Retained placenta		3	3		
- Obstructed labor		1	2		
- Post partum hemorrhage		1	0		
- Prolapsed umbilical cord		1	0		
Cesarean section rate	%	47	35	0.455°	n.s.
Female neonates	%	42	46	0.813°	n.s.
Birth weight	Grams	1,666	3,348	<0.001^*^	
APGAR at 5′ <7	%	21	12	0.367°	n.s.
pH art. <7.15	%	2	15	0.038°	
Transfer to NICU	%	94	8	<0.001°	

* t-test;

** *Man-Whitney-U-Test; °Fisher's Exact test; n.s., not statistically significant*.

The neonates' gender did not differ in both groups. Interestingly, no difference in the rate of newborns with a 5-min-Apgar lower than 7 was found in both groups and an arterial umbilical cord pH lower than 7.15 was even more frequent in the control group. Expectedly, referral to the Neonatology department occurred more frequently in the case group (Table 2). Likewise respiratory distress syndrome, neonatal infection and sepsis rates were higher in the case group newborns.

In vaginal smears, gram stain showed no statistically significant difference in the rate of abnormal flora/bacterial vaginosis (BV) (34) between the groups at T1 (onset of PPROM). At T2 (delivery) the abnormal flora/BV rate was significantly higher in the case group most likely due to ascending bacteria because of the state of ruptured membranes. At T3 no difference in the rate of abnormal flora/BV was found for both groups. The variety and number of bacteria found in cultures were increased in the case group at T1 and (possibly affected by the state of ruptured membranes) remained increased at T2 and even T3 (Table 3).

**Table 3:** Microbiota and histopathology.

	**Source**	**Legend**	**Timepoint 1 cases**	**Controls**	***p* =**	**Timepoint 2 cases**	**Controls**	***p* =**	**Timepoint 3 cases**	**Controls**	***p* =**
Gram stain	Vagina	% normal/abnormal/BV	51/33/16	74/9/17	n.s.	19/68/13	65/29/6	0.001	31/50/19	54/33/13	n.s.
Culture spec.	Vagina		5× *E.coli* 1× *Hämoph.inf*. 1× Gardnerella 1× Staphyloc. 1× *Strept.milleri* 6× *Mix.flora*	2× Gardner. 2× Mix.flora		5× *E.coli* 1× Enterococ. 1× Peptostrept. 1× Klebsiella 1× Enterobact. 1× Fungus 8× *Mix.flora*	5× Mix.flora		3× *E.coli* 2× *Strept.mill*. 2× Gardner. 1× Klebsiella 1× *Staph.aur*. 11× *Mix.flora*	1× *E.coli* 8× *Mix.flora*	
Candida	Vagina	*n* =	3	0	n.s.	0	1	n.s.	0	0	n.s.
GBS	Vagina	*n* =	5	1	n.s.	0	0	n.s.	3	2	n.s.
Mycoplasma	Vagina	*n* =	1	0	n.s.	1	0	n.s.	0	0	n.s.
Ureaplasma	Vagina	*n* =	11	8	n.s.	2	5	0.026	0	4	n.s.
Culture growth	Membranes	*n* =				12	7	n.s.			
Culture spec.	Membranes					4× *E.coli* 2× Enterococ. 3× Ureaplas. 1× *Hämoph.inf* 1× *Strept.vi*r. 1× Peptostrept. 1× Enterobact. 1× *Bact.fragil*. 3× *Mix.flora*	1× *E.coli* 1× Serratia 2× Ureaplas. 1× *Bact.fragil*.				
Histology	Placenta	% Chorioamnionitis				40	0	0.001	

*All p-values: Fisher's exact test. n.s., not statistically significant*.

Smears from the membranes showed again a larger number and variety of bacteria in the case group membranes (Table 3). Remarkably, in only one patient the same bacterium (Haemophilus influenzae) was found in the vagina at T1 and in the placenta/membranes at delivery (T2). Chorioamnionitis was diagnosed histopathologically significantly more often in the case placentae (Table 3).

Neither Chlamydia nor Gonococcus or Listeria were detected in all groups at all timepoints. Candida, group B streptococci, mycoplasma and ureaplasma colonization were not statistically different between both groups at all timepoints, except ureaplasma at T2 which was even higher in controls than in women with PPROM (Table 3).

Leucocyte count was significantly higher in the test group at T1 and T2, while 6 weeks after delivery at T3 there was no significant difference. Leucocytes increased from T1 to T2 in the test group, but not in the control group.

**Figure 1:**
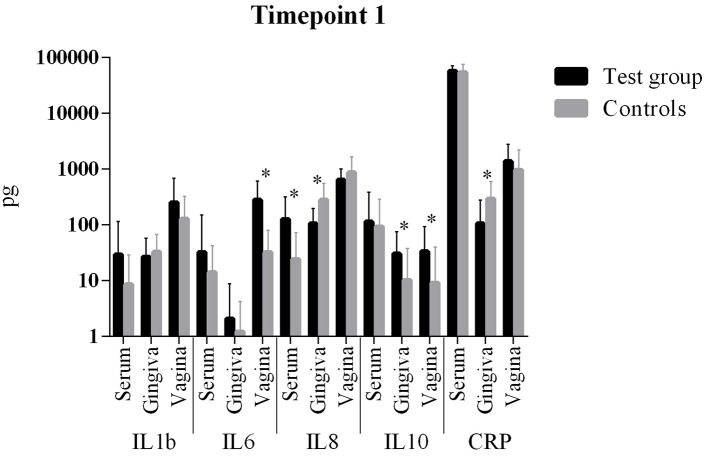
Comparison of cytokine levels in PPROM women (cases) vs. controls at timepoint 1. *statistically significant.

**Figure 2:**
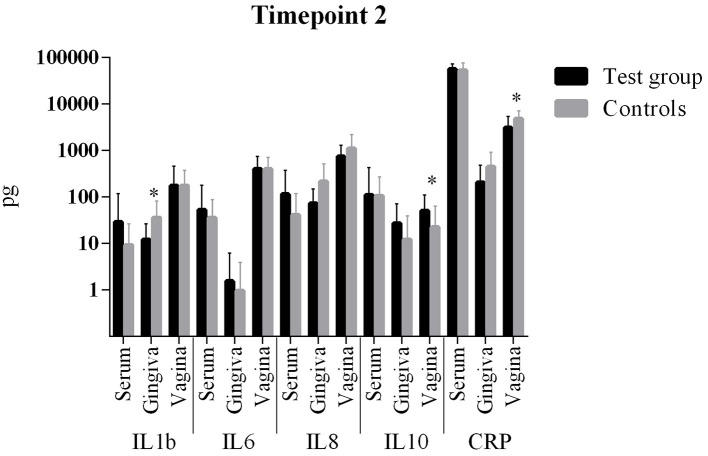
Comparison of cytokine levels in PPROM women (cases) vs. controls at timepoint 2. *statistically significant.

**Figure 3:**
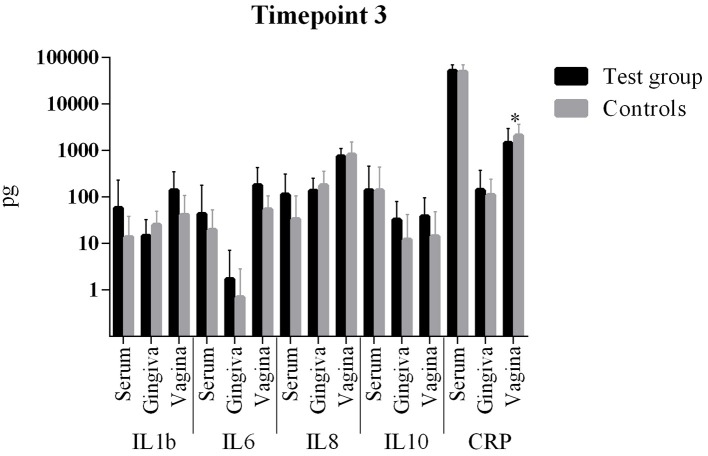
Comparison of cytokine levels in PPROM women (cases) vs. controls at timepoint 3. *statistically significant.

The comparison of cytokine levels in the three body compartments at the three timepoints showed (Table 4, Figures 1 – 3) at

- T1: The case patients had higher levels of pro-inflammatory IL-6 in the vagina and pro-inflammatory IL-8 in the blood, lower levels of IL-8 and CRP in the gingiva, and higher levels of anti-inflammatory IL-10 in gingiva and vagina
- T2: The case patients had increased levels of pro-inflammatory IL-1b in the gingiva, increased levels of IL-10 and a decreased CRP in the vagina
- T3: The case patients only showed lower CRP levels in the vaginal.

**Table 4:** Cytokines: comparison of case vs. control group at the 3 timepoints.

**Cytokine**	**Source**	**Cases vs. Controls**		
		**T1**	**T2**	**T3**
IL1b	Blood	n.s.	n.s.	n.s.
	Gingiva	n.s.	↓ (12 vs. 36)	n.s.
	Vagina	n.s.	n.s.	n.s.
IL6	Blood	n.s.	n.s.	n.s.
	Gingiva	n.s.	n.s.	n.s.
	Vagina	↑ (281 vs. 32)	n.s.	n.s.
IL8	Blood	↑ (126 vs. 24)	n.s.	n.s.
	Gingiva	↓ (105 vs. 280)	n.s.	n.s.
	Vagina	n.s.	n.s.	n.s.
IL10	Blood	n.s.	n.s.	n.s.
	Gingiva	↑ (30 vs. 10)	n.s.	n.s.
	Vagina	↑ (33 vs. 9)	↑ (50 vs. 22)	n.s.
CRP	Blood	n.s.	n.s.	n.s.
	Gingiva	↓ (105 vs 294)	n.s.	n.s.
	Vagina	n.s.	↓ (3,088 vs. 4,817)	↓ (1,442 vs. 2,070)

*↑:significantly higher levels; ↓ : significantly lower levels; n.s., not significant*.

*Brackets: Means in pg/ml. All p-values: Mann-Whitney-U-test*.

All other parameters in blood, vaginal and gingival fluid were not significantly different at the three timepoints (Table 4, Figures 1–3). However, from T1 to T2 IL-1b and IL-8 decreased in gingiva while IL-6 increased in the blood and CRP increased in the vagina. These interleukin patterns might be in line with the cascade theory: Inflammatory cytokines already start decreasing in the mouth while they increase in blood and vagina reflecting a chronological process starting in the oral cavity.

## Discussion

Our study focuses on the relationship between periodontitis and PPROM. Three different body compartments were therefore studied at three time points. The main finding is the difference in cytokine levels in different compartments in the case group at the time of PPROM as compared to controls. This means that an inflammatory process is in progress, and cytokine patterns suggest this to be initiated in the periodontal compartment, consecutively encroaching on further body compartments. However, this does not occur via bacterial spread since nucleic acid based methods and microbiologic cultures did neither reveal an association of oral and vaginal nor of vaginal and placental bacterial colonization.

Predicting and identifying women at risk for delivering preterm is challenging. A relation between periodontitis and APO has been suggested (11, 15), but the pathophysiologic link is not established. Oral inflammatory mediator changes have been reported in APO (8, 28, 35–37), but none of these studies focused on women with PPROM or cytokine levels in different body parts. A systematic approach examining systemic and local compartments might reveal pathophysiologic links between remote infection and PPROM.

The comparison of cytokine levels in the three body compartments at three timepoints showed significant differences between PPROM patients and controls. At the onset of PPROM the periodontal inflammation seems to already decline since pro-inflammatory IL-8 and CRP are decreased compared to controls and an immune response is already initiated as anti-inflammatory IL-10 (38, 39) reactively increases in the gingiva. At the same time pro-inflammatory IL-8 and IL-6, respectively, are increased in blood and vagina. Both cytokines are early markers of a beginning inflammation (40). This suggests that inflammation starts in the periodontal compartment and already begins to resolve, while in peripheral blood and vagina it is still ongoing in terms of an inflammatory cascade. From T1 to T2 the same situation is found: while inflammatory markers (IL-1b and IL-8) further decrease in the gingiva, IL-6 increases in the blood and both CRP and IL-10 increase in the vagina. The informative value of the differences in cytokine levels at T1 is emphasized by the condition that at 6 weeks post partum (T3) interleukin levels in both groups did not differ significantly. The inflammatory response seems to regress which is consistent with the systemic inflammatory hypothesis. The finding of an increased anti-inflammatory cytokine IL-10 in the vagina at T1 and T2 might be explained by the fact that rising IL-10 levels in the vaginal fluid reflect the initiation of the birth process (41, 42), besides being associated with intraamniotic infection (43).

The oral investigations' results showed an association between periodontal status and PPROM and oral periodontopathogens were more prevalent in the PPROM women (12). Importantly, although periodontopathogenic bacteria were detected in vaginal samples, in none of the women these bacteria were found in both the vagina and the mouth concomitantly (12), corroborating the hypothesis that not bacterial spread but systemic inflammation might be a contributing factor in developing PPROM. This is further supported by the finding that abnormal flora and bacterial vaginosis were equally frequent in both the cases and the controls, respectively, at the occurrence of PPROM (timepoint 1). Vaginal bacterial dysbalance seems not to be the exclusive cause for PPROM. The variety and number of bacteria found in cultures was increased in the case group at T1, but remarkably in only one patient the same bacterium has been affirmed in both the vagina at T1 and the placenta smears at T2 (delivery), which again is in conflict with the hypothesis that ascending infection is the primary mechanism for PPROM.

Recent findings showing that the placental microbiome profile is much more akin to the oral microbiome than to the lower genital tract (24) do not support the theory that ascending infection is the primary cause of PPROM. Likewise, microbiome profile at PPROM does not correlate with latency duration supporting our notion that systemic inflammation may rather play a role in the onset of PPROM than microbiota dysbalance (44). Furthermore, direct spread of oral bacteria to the reproductive tract is unlikely since PTB is rarely associated with intrauterine infection with oral flora (22), and bacteria are mostly found in term membranes without causing PTB (1, 23). In our patients, periodontopathogens found in the gingiva have not been verified in the placenta/membrane swabs (12) and no difference was found in the rate of abnormal flora and bacterial vaginosis between cases and controls at the time of PPROM. Moreover, in only one patient one of the bacteria found in the vagina at T1 was verified in the placenta/membranes at T2. All these findings argue against the theory of direct spread of bacteria as the causative mechanism of PPROM. Accordingly, recent studies have shown the gut microbiome being linked to several diseases and although the exact mechanism is not fully understood a systemic cytokine release has been demonstrated and made accountable for disease occurrence (45, 46). A more extensive investigation of the microbiome-inflammation-interaction in different body compartments is desirable.

A strength of our study is the unique study design with a comprehensive approach including simultaneous examination of different body compartments, microbiological and nucleic acid based assessment and comparison of inflammation markers, in parallel with clinical periodontological assessment at different timepoints. The use of the multiplex method is considered particularly convenient (47).

A weakness is that we have no data of women at “T0,” i.e., before PPROM becomes clinically evident. In particular, an early timepoint for analysis in the first trimester or even before onset of pregnancy would be valuable in order to have the whole picture and understand the mechanism how periodontitis may cause PPROM and APO. Unfortunately, due to the low incidence of PPROM and the high expenditure such a prospective study would be difficult to realize. The lack of published data and the complexity of recruiting women for examination at three time points impeded a sound power analysis and inclusion of a large number of patients. Thus, the limited number of women in our study might explain why differences have not been observed among some of the measured parameters, and maternal plasma cytokine concentrations are difficult to interpret in APO patients (48). Moreover, paper points were used for samplings of subgingival plaque. Since they do not allow to collect the biofilm but only planktonic bacteria, use of a courette might have been the better choice for sample collection.

In summary, the results of our study can be interpreted in line with our initial hypothesis that systemic inflammation—initiated and triggered by periodontal disease—might play a role in developing PPROM. Bacterial spread or ascension seem not to represent the exclusive pathophysiologic cause for PPROM. There is a systemic inflammation in progress at the time of PPROM and we speculate that the inflammatory pathway rather than ascending infection alone contributes to PPROM and—consecutively—PTB.

## Data Availability Statement

The raw data supporting the conclusions of this manuscript will be made available by the authors, without undue reservation, to any qualified researcher.

## Ethics Statement

The studies involving human participants were reviewed and approved by Ethics Committee of the Canton of Bern (Nr. 091/10). The patients/participants provided their written informed consent to participate in this study.

## Author Contributions

SM: project development, data management, data analysis, manuscript writing, and approval of the final manuscript. SA-M: data collection, data interpretation, and approval of the final manuscript. PS: data collection, data analysis, and approval of the final manuscript. AS and RP: project development and approval of the final manuscript. SE: data management and approval of the final manuscript. DS: project development, manuscript editing, and approval of the final manuscript.

### Conflict of Interest

The authors declare that the research was conducted in the absence of any commercial or financial relationships that could be construed as a potential conflict of interest.
